# The New York Code of Ethics

**Published:** 1882-05

**Authors:** 


### THE NEW YORK CODE OF ETHICS.

From the profession of almost every state and section, as reflected by the medical journals, come the earnest protests against the attempted innovation of the New York State

Medical Society upon the code of ethics. Some of the comments are extremely vigorous and some even caustic. The unanimity of opinion, however, is remarkable. This is especially noticeable in the Southern and Western States. It gives us renewed faith in the honor and integrity of the profession. A few journals, chiefly in the large cities, defend, or mildly apologize, for the action of the society; but the defense of a weak cause must always be itself weak.

